# Improving systematic rabies surveillance in Cameroon: A pilot initiative and results for 2014-2016

**DOI:** 10.1371/journal.pntd.0006597

**Published:** 2018-09-06

**Authors:** Casimir Ledoux Sofeu, Anaïs Broban, Amadou Njifou Njimah, Jean Blaise Momo, Serge Alain Sadeuh-Mba, Sophie Druelles, Maïna L’Azou, Mathurin Cyrille Tejiokem

**Affiliations:** 1 Epidemiology and Public Health Service, Centre Pasteur du Cameroun, Member of the International Network of Pasteur Institutes, Yaoundé, Cameroon; 2 Global Epidemiology, Sanofi Pasteur, Istanbul, Turkey; 3 Mbouda Health District Hospital, West Regional Delegation of Public Health, Ministry of Public Health, Bafoussam, Cameroon; 4 Faculty of Medicine and Pharmaceutical Sciences, University of Douala, Douala, Cameroon; 5 Virology Service, Centre Pasteur du Cameroun, Member of the International Network of Pasteur Institutes, Yaoundé, Cameroon; 6 Global Epidemiology, Sanofi Pasteur, Lyon, France; Universidad Nacional Mayor de San Marcos, PERU

## Abstract

Canine rabies is endemic in Cameroon, but human rabies exposures and cases are likely underreported because of inadequate surveillance. In 2014, the surveillance network in the West region of Cameroon was reinforced by introducing a new anti-rabies center, a framework for data collection and evaluation, provisions for sample collecting and laboratory confirmation, and training for health professionals. The objective of this observational cohort study was to describe the incidence and characteristics of reported exposures and human and animal rabies cases following this reinforcement of the existing rabies surveillance system. The surveillance network consisted of local, regional, and national health and veterinary authorities in 11 of the 20 West region districts, and was completely integrated within the existing national rabies surveillance network. Animal exposures and suspected rabies exposures, the suspected rabid animals involved, and laboratory confirmation of human and animal rabies cases were recorded in a centralized information database. Between January 2014 and June 2016, the network recorded 1340 animal exposure cases for an overall incidence rate of 38.2 animal exposures per 100,000 people, four confirmed rabies-positive animals, and one confirmed human rabies case out of four clinically suspected cases. In contrast, 62 animal exposures and an overall incidence rate of 6.1 exposures per 100,000 people were reported for the West region districts not participating in the reinforced surveillance. Of the 925 animal exposure victims for whom a detailed case report form was completed, 703 were considered to be at risk of rabies and only 428 (61%) of these received any post-exposure prophylaxis in the form of rabies vaccine. Obstacles encountered within the network included low rates of animal sample submission and animal follow-up by veterinarians. Reinforced rabies surveillance in the West region of Cameroon has provided the most accurate estimate of the region’s disease and exposure burdens to date, and indicates that animal exposures are substantially underreported. The reinforced network also signaled that greater access to post-exposure prophylaxis is needed. Integration of regions not covered by the surveillance network and efforts to improve engagement of veterinary services will be needed to reveal the true burden of rabies in Cameroon.

## Author summary

Dog-mediated human rabies causes tens of thousands of human deaths annually in economically disadvantaged countries. The disease is almost invariably fatal once symptoms appear but can be prevented if post-exposure prophylaxis is received within a few days after the exposure. Canine rabies is endemic in Cameroon but inadequate surveillance has meant the extent of the disease is unknown and underappreciated. In 2014, reinforcements were made to the rabies surveillance network in the West region of Cameroon. The reinforced surveillance network was formed of local, regional, and national health and veterinary authorities and collected information related to suspected rabies exposures, prophylaxis administration, and outcome. This study described the findings from the reinforced network and provided the most reliable estimate of the region’s disease burden to date. The study also highlighted that improved access to life-saving prophylaxis is needed for bite victims of suspected rabid animals. The findings from this study will help implement a new nationwide surveillance system in Cameroon.

## Introduction

Human deaths from rabies persist in low- and middle-income countries where the disease is endemic in animal populations [1,2]. Bites from infected domestic dogs account for over 99% of the 59,000 human rabies cases estimated worldwide per year [2,3], most of which occur in Asia and Africa [4–6]. Humans bitten by rabid animals must immediately receive post-exposure prophylaxis (PEP) to prevent disease development, which has a case-fatality rate of almost 100% [1,2,7].

Canine rabies is endemic in Cameroon [8–10], with the highest reported incidence in the Center, West, and Littoral regions [8]. Between 1990 and 1999, a retrospective study reported a mean (± standard deviation) of 43 ± 13 human rabies deaths per year [8]. However, the true burden of human rabies cases and exposures in the country is unknown. Estimates of human rabies cases and deaths in Cameroon have varied widely [8,10,11] and are probably inaccurate, especially in rural areas where healthcare access is limited. Moreover, many bite victims may not seek PEP due to poor awareness of the disease or its prevention using PEP or because of associated financial costs, all of which contribute to underreporting [5].

Animal and human rabies have been notifiable diseases in Cameroon since 2001; all animals suspected of rabies must be quarantined and reported to local and national veterinary authorities, and since 2014, bites from suspected rabid animals are reported weekly by district health centers to the regional health delegations [12]. Rabies control legislation also requires vaccination of pet dogs and cats and requires that owners of biting animals are recorded in each district. Although rabies control efforts, such as yearly reduced-price pet vaccination events, radio information campaigns, and dog culling, exist in Cameroon, the impacts of these programs are unknown [9,13].

Improved rabies surveillance networks are recognized as a key strategy to tackle underreporting of rabies cases and exposures [3,14–17] and are a cornerstone of the strategic global plan proposed by the World Health Organization (WHO), the World Organisation for Animal Health, the Food and Agriculture Organization of the United Nations, and the Global Alliance for Rabies Control to end canine-mediated human rabies by 2030 [15,18,19]. To achieve this goal, existing rabies control and PEP distribution strategies need to be strengthened and resourced appropriately, and new programs need to be established in areas where animal rabies is endemic [15]. In Cameroon, the infrastructure to survey and manage potential rabies exposures has been limited by suboptimal funding, poor coordination, inadequate training, and lack of sample collection and submission. New measures from the Cameroon Ministry of Public Health are beginning to tackle some of these limitations through new regional anti-rabies centers (ARCs) that have improved PEP access and availability.

In 2014, the Centre Pasteur du Cameroun (CPC) and Sanofi Pasteur helped reinforce the surveillance network in the West region of Cameroon. The reinforced network was implemented as a regional model to validate current surveillance approaches, and to identify and solve obstacles to rabies surveillance. Here, we describe the reinforced network’s operation and performance, and the results obtained between January 2014 and June 2016.

## Methods

### Study area and design

**Fig 1:**
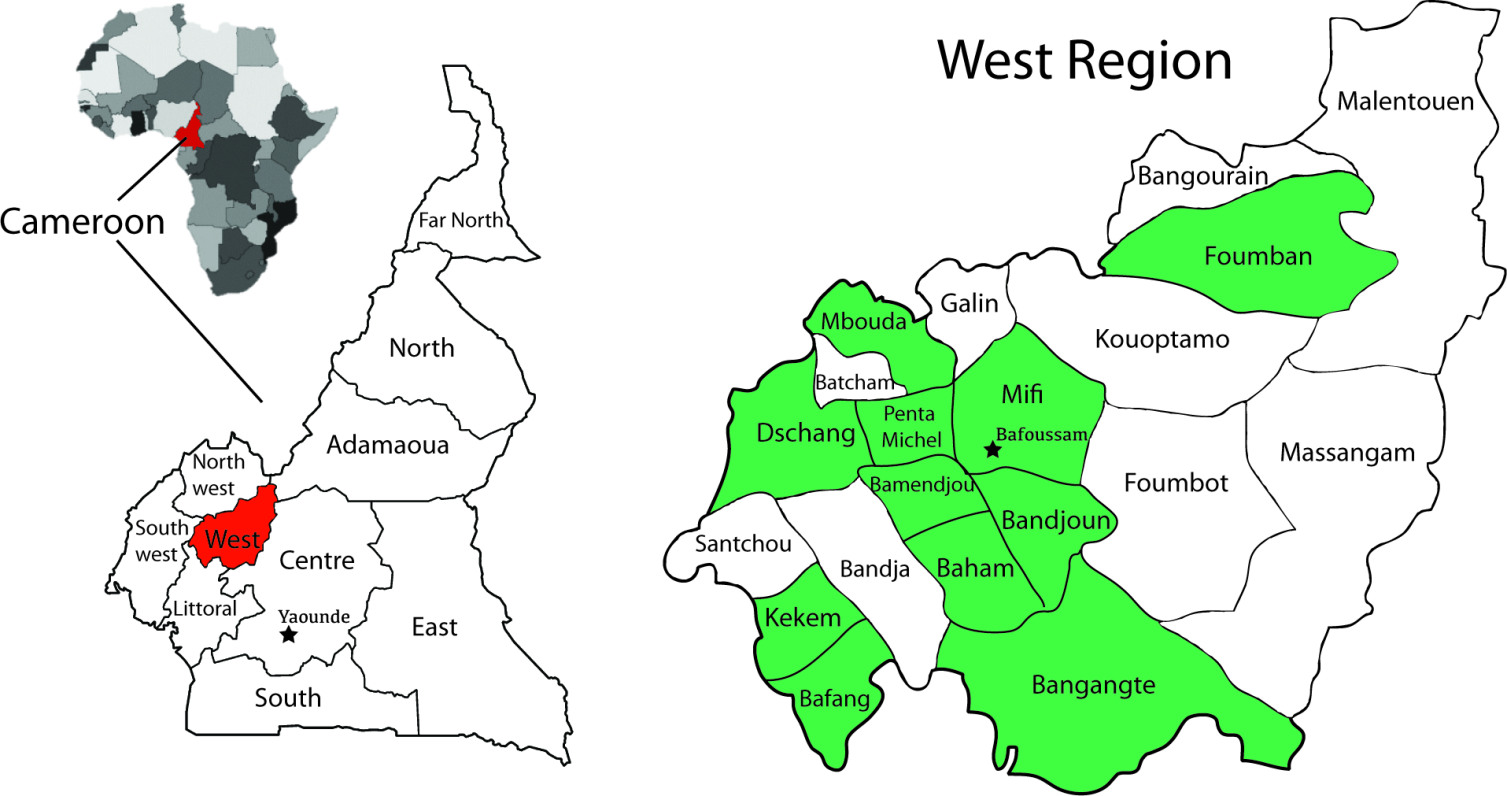
Geographical location of the reinforced surveillance network and participating health districts. The reinforced surveillance network operated in the West region of Cameroon between January 2014 and June 2016. The surveillance network was managed by the Centre Pasteur of Cameroon, Yaoundé, and included 11 of the West region’s 20 health districts (indicated by green shading). Post-exposure prophylaxis (PEP) was available to exposed individuals at the ARC in Bafoussam and at some pharmacies throughout the region.

This was a prospective observational study performed in 11 of the 20 health districts in the West region of Cameroon from January 2014 to June 2016 (**Fig 1**). The estimated population of the West region was 1,810,648 inhabitants in 2011. The objective of the study was to describe the incidence and characteristics of animal exposures and rabies cases in these districts following reinforcement of the existing rabies surveillance system.

### Ethics statement

This was a national public health surveillance activity approved by the Cameroonian Ministry of Public Health and reviewed by the National Ethics Committee. Approval by an institutional review board or written informed consent was not required. Data concerning the exposed humans and animal owners were anonymized before analysis.

### Rabies surveillance system and adopted reinforcement measures

The existing rabies surveillance network in Cameroon was formed of district public health and veterinary units that reported human and animal rabies cases to the regional delegations. These then compiled the regional findings and reported them to the appropriate national government ministries. Before the reinforcement measures were adopted, most animal exposures and animal and human cases were not notified, surveillance was mostly passive and incomplete, and no case report forms (CRF) or a rabies case register were available. Animal exposure victims who were aware of the risk of rabies could seek PEP in Yaoundé, the capital city, or purchase rabies vaccine at a local pharmacy if it was available and they could afford to buy it.

In 2013, the existing rabies surveillance network was reinforced in 11 of the 20 health districts in the West region of Cameroon, covering a population of 1,403,960 people (**Fig 1**). The remaining nine districts were not included because of study budget limitations, low populations, long distance from Bafoussam, or anticipated difficulties in surveillance data collection. The population of these districts is approximately 406,688.

The reinforcements made to the network included establishing an ARC in the West region’s principal city, Bafoussam, in late 2013; providing supplies and equipment to local units for collecting, preserving, and transporting animal and human samples; and supplying case registers and CRFs to healthcare professionals and veterinarians in each district. A technician was employed at the CPC in Yaoundé to manage the network, perform sample analysis, and to collect and confirm (by weekly phone call) epidemiological data from each district. Computing resources were supplied to a data manager based at the CPC who was responsible for maintaining a database of collected information. All communication fees and costs for sample collection and transportation were supported, and reagents and consumables were provided to support the centralized laboratory activities in the CPC. Training was provided for ~45 people including each district’s chief medical officer, emergency ward personnel, a chief veterinarian from each health district, and members of the West regional health delegation. The training covered the network organization; human and animal rabies case recognition and definitions; rabies surveillance network procedures and case notification; handling of animal exposure cases; sample collection, storage, and transfer; and pharmacovigilance. Resources were also put into raising awareness among healthcare and veterinary professionals through refresher training and among the public through leaflets, advertisements, media announcements, and participation in World Rabies Day and other conferences.

During the study, each of the 11 health districts received yearly follow-up visits by the study coordinators to ensure coherence and good practice, and adjustments were made where required. Yearly study progress meetings also took place to present interim collected findings, refresh training, and discuss difficulties.

### Management of animal exposures and suspected human rabies cases

**Fig 2:**
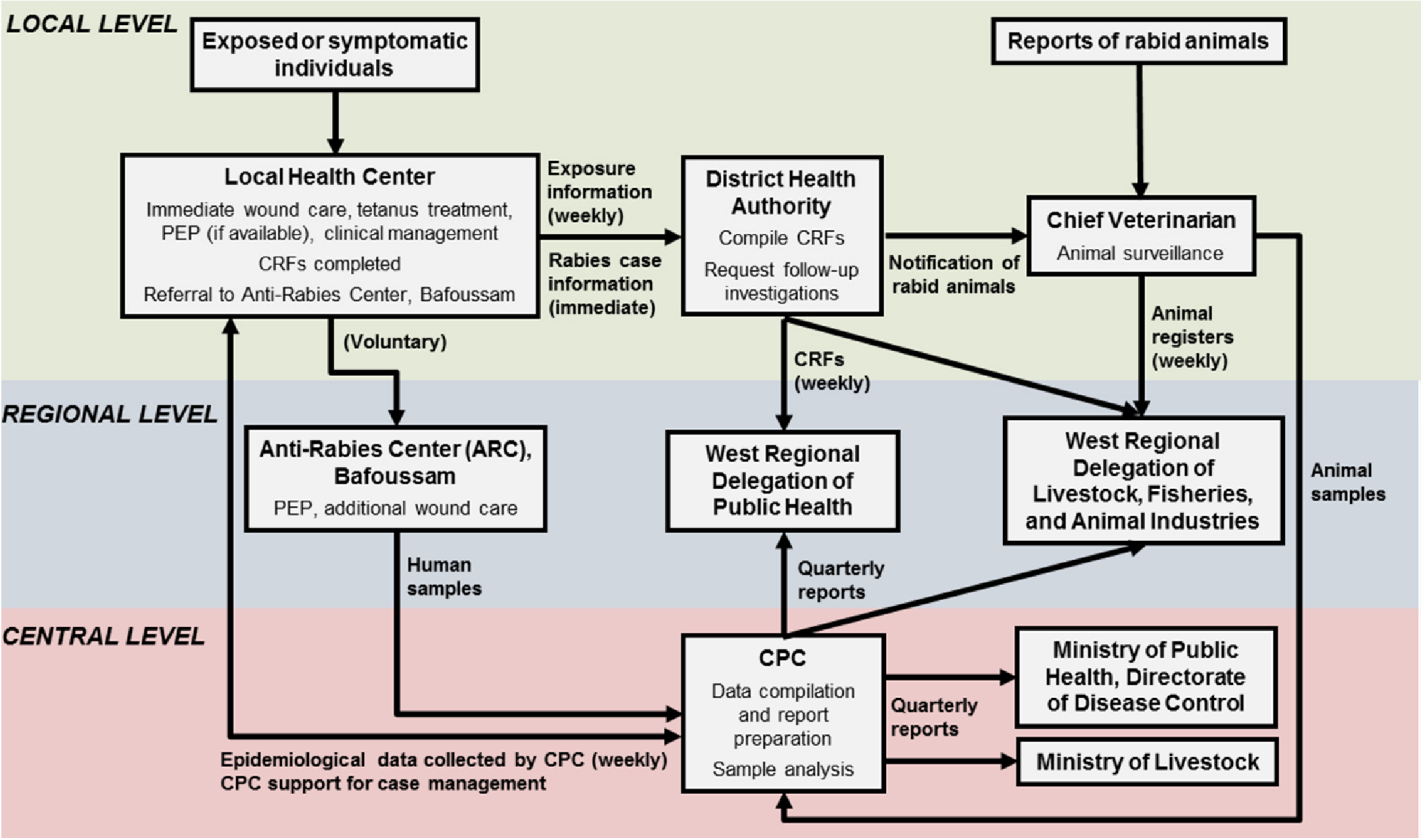
Structure of the reinforced surveillance network in Cameroon. Abbreviations: CPC, Centre Pasteur du Cameroun; CRF, case report form.

The structure and operation of the reinforced rabies surveillance network is presented in **Fig 2**. All animal exposure victims who presented at a local health authority were included in the study. Exposed individuals had any wounds cleaned and may have received anti-tetanus medication according to their vaccination status and willingness. Healthcare personnel completed a CRF with the victim or victim’s relatives to collect information regarding the wound; animal involved; socio-demographic data of the victim; the exposures, PEP, and immunizations received prior to the current exposure; and the wound treatment received (rabies-specific and non-specific). Whether an animal exposure was at risk of transmitting rabies was determined by the medical staff based on information collected from the victim and/or the victim’s family, and the animal owner, if available. Globally, an animal was considered at risk of transmitting rabies if it had not been vaccinated or its vaccination status was unknown, the owner was unknown, the animal had disappeared or died after the attack, or the animal had behaved abnormally, i.e. had displayed any of the signs of rabies listed in the definition of animals suspected to be rabid [20]. However, staff were also aware that such determinations of animal behavior made by untrained persons could be unreliable. Thus, a conservative approach was used in some cases due to the type and severity of the exposure combined with the fact that rabies is endemic in Cameroon. This information was enough for the medical staff to recommend PEP or not. Exposure victims determined to be at risk of rabies due to a category II or III exposure from an animal of unknown or uncertain health status or an animal suspected to be rabid, as well as victims who requested PEP despite not being considered at risk, were then either referred to the ARC in Bafoussam where they could receive PEP directly, or were instructed to purchase and receive PEP locally. PEP was administered by intramuscular injection according to the Zagreb (recommended) or Essen regimen [21,22]. Additional wound care (e.g., additional cleaning and antibiotics) was administered if necessary. Loss to follow-up after PEP administration was recorded as ‘information missing’.

Individuals suspected to have rabies were clinically managed by the chief doctor and nurses of each health district. Individuals presenting at local health centers were suspected to have rabies if they had been in contact with a suspected rabid animal and had at least one of the following signs: headache, cervical pain, nausea, fever, hydrophobia, anxiety, agitation, abnormal tingling sensations or pain at a bite site [20]. In each suspected rabies cases, three saliva samples collected at 3 hours apart and a skin biopsy from the nape of the neck were taken. These specimens were accompanied by a human rabies CRF containing details about the samples taken, patient demographics, type of wound or exposure, clinical signs, and information about the animal involved. The samples were maintained at −20°C and transported on ice (with the human rabies CRF) to the CPC for rabies confirmation within 48 hours. On arrival, samples were analyzed immediately or stored at −80°C when immediate processing was not possible. Each sample was screened for the detection of rabies virus using both a reverse-transcription (RT), hemi-nested polymerase chain reaction (PCR) as described previously [23] and a SYBR green-based quantitative RT-PCR -targeting coding regions of the polymerase and nucleocapsid genes, respectively [24].

Each of the 11 included health districts compiled and sent CRFs weekly to the West Regional Delegation of Public Health, where records were entered into a centralized computer database. In addition, the CPC contacted each local health center in the study districts weekly to obtain epidemiological information and the number of exposures recorded locally during the previous week.

### Animal rabies case and sample management

For each animal exposure case, where possible, medical staff referred the owner of the involved animal to contact the chief veterinarian for examination and follow-up, but did not necessarily contact the veterinarian directly. In response, and when possible, the local veterinary service then completed a register recording the owner’s details and the animal’s species, age, sex, anti-rabies vaccination status, behavioral history, and current health status, and organized the follow-up visits for animals with unclear health status or those considered to be at risk of being rabid. After an at-risk exposure, follow-up observations (if performed) were conducted for 10 days for dogs or cats or for 15 days for other animals. During that period, one visit was to collect baseline data, a second was for an initial report, and a third was for the final report. However, veterinary follow-ups were limited due to excessive workloads, lack of sufficient facilities to maintain and confine animals under observation, and by the unwillingness of animal owners to pay for the surveillance/observation, which is a requirement in Cameroon. On observation, an animal was clinically suspected of rabies if it displayed any of the following signs: unprovoked aggression, foaming at the mouth, paralysis, incoordination, hoarse bark, hydrophobia, weakness, seizures, or loss of appetite [20]. Registers were compiled and sent weekly to the West Regional Delegation of Livestock, Fisheries, and Animal Industries. The chief veterinarian was also responsible for overseeing collection and transfer of animal head samples to the CPC for analysis and for performing follow-up investigation of laboratory-confirmed rabies cases. Veterinarians were not paid for animal sample collection but shipping supplies (cooler and packing materials, ~30€) and costs (sample shipment, ~8€ and cooler return, ~2€) were provided by the project.

Within 48 hours of receiving animal head samples at the CPC, a necropsy was performed and at least two regions of the brain were tested for rabies virus using the fluorescent antibody test [25] with rabbit IgG against rabies virus nucleocapsid (Bio-Rad, Marnes-la-Coquette, France). Samples that were negative in the fluorescent antibody test were crushed and inoculated onto murine neuroblastoma cell cultures [26] to confirm absence of rabies virus, as previously described [25]. Results obtained by the CPC were reported back to the chief veterinarian, the appropriate district’s health authority, the directorates of disease control at the Ministry of Public Health, and the Ministry of Livestock, Fisheries, and Animal Industries.

### Case follow-up

Follow-up investigations with family members were performed subsequent to any human rabies death from suspected or confirmed rabies or any case of laboratory-confirmed animal rabies. The purpose of the investigations was to determine and control additional human or animal exposures. In such cases, the implicated district’s health authority and the ministries in charge of human and animal health were immediately contacted and notified of the human case or laboratory-confirmed animal case. Follow-up investigations were also performed in cases where high numbers of persons were exposed to a confirmed rabies case. Follow-up investigations were conducted by local and central health and veterinary authorities.

### Data management and analysis

Information reported from the district authorities was regularly reviewed at the CPC for missing or incoherent entries and followed-up at the source if necessary to collect missing data. Follow-up visits at the West Regional Delegation of Public Health in Bafoussam were performed 2−3 times per year to monitor data management. Statistical analyses were descriptive and missing values were not excluded. Microsoft Access 2007 software was used to manage the database. Statistical analysis was performed using R version 2.15 [27].

Surveillance reports of the West region were disseminated three times per year to the Directorate of Disease Control of the Cameroon Ministry of Health, the Ministry of Livestock, and the health and livestock authorities of the West region.

## Results

### Exposures and animal surveillance

Between January 2014 and June 2016, a total of 1,402 animal exposures were reported in the West region of Cameroon. Of these, 925 exposures (66.0%) had completed CRFs and were from the 11 health districts included in the reinforced surveillance network (**Table 1**). An additional 415 exposures were recorded from weekly follow-up phone calls to the health centers in the study districts, for a total of 1340 exposures in the study districts. However, little information was available for these additional exposures because no CRFs were completed for them. A total of 62 other exposures were reported to the CPC from other or unknown districts in the West region. Over one-half of all exposures were recorded at the ARC in Bafoussam, in the Mifi district. Annual exposure rates in the study districts ranged from a low of 8.7 per 100,000 in Foumban to a high of 77.9 per 100,000 in Bafoussam. By contrast, only 62 exposures were recorded for all the districts not included in the study, and the overall exposure incidence rate for these districts was 6.1 per 100,000. Exposures did not show any seasonality (Figure A in S1 Information), but annual exposure totals and incidence rates did tend to increase during the surveillance period.

**Table 1:** Numbers of animal exposures reported within districts of the West region of Cameroon between January 2014 and June 2016.

		Number of exposures					Annual incidence per 100,000			
Location (department)	Population^a^	2014	2015	2016^b^	Total	%	2014	2015	2016^b^	Mean^c^
Bafang (Haut-Nkam)	92,651	8	5	24	37	4.0	8.6	5.4	51.8	16.0
Bafoussam ARC (Mifi)	247,925	166	217	100	483	52.2	67.0	87.5	80.6	77.9
Baham (Hauts-Plateaux)	49,823	11	4	7	22	2.4	22.1	8.0	28.0	17.7
Bamendjou (Hauts-Plateaux)	48,437	13	5	10	28	3.0	26.8	10.3	41.2	23.1
Bandjoun (Koung-Khi)	111,948	20	11	2	33	3.6	17.9	9.8	3.6	11.8
Bangangté (Ndé)	110,979	44	30	12	86	9.3	39.6	27.0	21.6	31.0
Dschang (Ménoua)	206,652	19	13	23	55	5.9	9.2	6.3	22.2	10.6
Foumban (Noun)	166,411	10	10	16	36	3.9	6.0	6.0	19.2	8.7
Kekem (Haut-Nkam)	38,105	13	11	0	24	2.6	34.1	28.9	0.0	25.2
Mbouda (Bamboutos)	244,846	30	31	19	80	8.6	12.3	12.7	15.6	13.1
Penka-Michel (Ménoua)	86,183	3	30	8	41	4.4	3.5	34.8	18.6	19.0
Total documented exposures, study districts only	1,403,960	337	367	221	925	100.0	24.0	26.1	31.4	26.4
Additional exposures from phone follow-ups (no CRF), study districts only^d^		143	186	86	415	−				
Total exposures, study districts only	1,403,960	480	553	307	1340	−	34.2	39.4	43.7	38.2
Exposures documented from other or unknown districts^e^	406,688	27	28	7	62	−	6.6	6.9	3.4	6.1

ARC, anti-rabies center; CRF, case report form

^a^ District populations of the West region of Cameroon in 2011. Source: Cameroon West region health districts.

^b^ January to June (week 26).

^c^ Calculated for 2.5 years.

^d^ Exposure notifications recorded by phone follow-up to health centers in the 11 study districts; no CRFs or additional information was recorded for these exposures.

^e^ Exposures reported from other or unknown districts, with CRFs.

Dogs were the most common species involved in the exposures, responsible for 77.6% (718/925) of cases (**Table 2**). The animals involved were considered to be at risk of transmitting rabies in 68.9% (637/925) of cases. Most animals (64.0%; 592/925) were known or had a known owner, most (65.8%; 609/925) were still alive at consultation, and only 12.6% (117/925) were reported to have been vaccinated. Only 14.4% of the animal exposure cases were followed-up with a visit by a veterinarian.

**Table 2:** Characteristics of animals involved in documented exposures.

	Total (N = 925)	
Characteristics	n (%)	
Species		
Dog	718 (77.6)	
Cat	22 (2.4)	
Pig	12 (1.3)	
Mouse	6 (0.6)	
Other species	11 (1.2)	
Information missing	156 (16.9)	
At risk of rabies?^a^		
No	222 (24.0)	
Yes	637 (68.9)	
Information missing	66 (7.1)	
Animal has an owner?		
No	260 (28.1)	
Yes	592 (64.0)	
Information missing	73 (7.9)	
Animal status at time of consultation		
Alive	609 (65.8)	
Disappeared	207 (22.4)	
Killed	29 (3.1)	
Found dead	11 (1.2)	
Information missing	69 (7.5)	
Animal vaccinated?		
No	458 (49.5)	
Yes	117 (12.6)	
Information missing	350 (37.8)	
Follow-up visit(s) made by veterinarian?		
No	605 (65.4)	
Yes	133 (14.4)	
Information missing	187 (20.2)	

^a^ An animal was considered at risk of being rabid if it had not been vaccinated, if the owner was unknown, if the animal had disappeared or died after the attack, or if the animal had behaved abnormally, i.e. had displayed any of the signs of rabies.

Between January 2014 and June 2016, animal samples from six dogs and one pig were collected during veterinary follow-up investigations and submitted for rabies diagnosis. Four of the dog samples were confirmed positive for rabies.

### Post-exposure clinical management

Animal exposure victims were female in 53.8% of cases and the median age was 20 years (**Table 3**). Most reported exposures consisted of animal bites (91.4%) or scratches (7.4%), and were generally cutaneous exposures (88.9%). The median delay before consultation was 1 day (range, 0−3 days). Of the 925 animal exposure victims presenting at healthcare units, 703 were considered to have been exposed to an animal at risk of rabies or of unknown status and were recommended for PEP; 428 (61%) of these victims received at least some of the required PEP vaccinations and 275 (39%) did not receive any. Of all persons who began the PEP vaccination series, 90 (17.4%) chose to receive PEP despite being considered not at risk of rabies and 282 (54.4%) were confirmed to have completed the PEP series. Thus, at least 146 (34%) of the victims considered to be at risk of rabies and who began PEP did not complete it. Overall, at least 421 (60%) of the exposure victims considered to be at risk of rabies either did not receive any PEP or did not receive all PEP vaccinations. Nearly all patients who began PEP (496/518; 95.8%) received it by the Zagreb protocol [21]. No adverse events to PEP were reported. Most of the 925 exposure victims received some form of tetanus prophylaxis (669 [72.3%] received anti-tetanus serum; 198 [21.4%] received anti-tetanus vaccine) and most (624; 67.5%) received antibiotics (Table A in S1 Information).

**Table 3:** Characteristics of animal exposure victims and treatments received.

Characteristic	Value (N = 925)
Sex, n (%)	
Female	498 (53.8)
Male	416 (45.0)
Information missing	11 (1.2)
Age, years^a^	
Median (IQR)	20 (9–42)
Age category, n (%)^a^	
> 15 years of age	529 (57.2)
≤ 15 years of age	390 (42.2)
Information missing	6 (0.6)
Type of exposure, n (%)	
Bite	845 (91.4)
Scratches	68 (7.4)
Licking	2 (0.2)
Information missing	10 (1.1)
Exposure site, n (%)	
Cutaneous	822 (88.9)
Mucous	6 (0.6)
Information missing	97 (10.5)
Delay (days) between animal bite and medical consultation^b^, median (IQR)	1 (0−3)
Previous history of exposure to suspect rabid animal, n (%)	
No	822 (88.9)
Yes	73 (7.9)
Information missing	30 (3.2)
PEP administered, n (%)	
Yes	518 (56.0)
Animal at risk of rabies or unknown^c^	428 (86.6)
Animal not at risk of rabies^c^	90 (17.4)
No	407 (44.0)
Animal at risk of rabies or unknown^d^	275 (67.6)
Animal not at risk of rabies^d^	132 (32.4)
PEP protocol used, n (%)^c^	
Zagreb	496 (95.8)
Essen	15 (2.9)
Information missing	7 (1.3)
PEP completed, n (%)^c^	
Yes	282 (54.4)
Information missing^e^	236 (45.6)

IQR, interquartile range; PEP, post-exposure prophylaxis.

^a^ N = 919

^b^ N = 823

^c^ Calculated percentage of people treated

^d^ Calculated percentage of people not treated

^e^ It can be assumed that these individuals did not complete PEP, but this cannot be confirmed.

### Human rabies cases

In 2014 and 2015, four samples from suspected human rabies cases were submitted for laboratory testing in the West region districts participating in the reinforced surveillance, of which one was confirmed positive for rabies. Seven samples were submitted from the rest of the country, of which three were confirmed positive. No human rabies samples were submitted for rabies confirmation between January and June, 2016. The confirmed case in the West district was an 8-year-old boy who presented with symptoms of furious rabies and subsequently died (**Table 4**). Of the three suspected cases that were not confirmed for rabies, it remains possible that the two that died were also rabies cases, whereas the one that survived was probably not a rabies case.

**Table 4:** Outcomes of confirmed and suspected human rabies cases in the West region, 2014−2016.

Sex	Age (years)	Symptoms/signs	Type of sample	Result	Outcome
Male	8	Fever, agitation, hydrophobia, exposure to suspected rabid dog	Saliva/skin biopsy	Confirmed	Died
Male	8	Information not available	Skin biopsy	Not confirmed	Died
Male	5	Information not available	Skin biopsy	Not confirmed	Died
Male	15	Agitation, anxiety, logorrhea, history of animal bite	Saliva/skin biopsy	Not confirmed	Survived

## Discussion

This study showed that reinforcements made to the existing Cameroon rabies surveillance system can improve rabies reporting, which ultimately allows for better estimates of the true rabies burden. In 2014, the first year of the reinforced surveillance network, 337 fully documented animal exposures (with CRFs) and 143 undefined exposures (no CRFs) were recorded in the study districts for a total of 480 exposures. Similarly high numbers of animal exposures were also recorded for 2015 (553) and for 6 months of 2016 (307). These numbers for approximately 78% of the West region population are 8−10-fold greater than the 57 exposures (without CRFs) previously reported for the entire West region in 2013 [28]. In addition, annual exposure incidence rates in the study districts were approximately 4-fold higher than those in the West region districts not participating in the reinforced surveillance. Together, these findings suggest that exposures were being underreported in the West region, as well as in other regions of Cameroon covered by the existing surveillance systems. Our findings suggest that if similar reinforcements can be implemented throughout Cameroon, the burden of rabies could be more precisely defined at the national level.

Animal exposure incidence rates exhibited some geographic and temporal variations. By far, the highest numbers of exposures and incidence rates were recorded at the ARC in Bafoussam. This is likely due to a high local level of rabies awareness due to the presence and activities of the ARC in this community. The ARC distributes information about rabies, provides a high level of care for exposure victims, and provides direct access to PEP. Exposure victims also come to the ARC from other districts, either directly or as referrals, but the surveillance system records those exposures for the districts in which they occurred. In general, animal exposure numbers and incidence rates tended to increase during the study period. We consider this to be an indication that surveillance measures and notifications were becoming more efficient throughout the system rather than a reflection of authentic increases in animal exposures across most districts. The trend could also mean that community awareness of rabies was also increasing.

Despite the improved surveillance, the incidence of animal exposures and the numbers of human rabies cases are still far lower than previously reported estimates for Cameroon [3]. By comparison, Hampson et al estimate a dog bite incidence of 128/100,000 for most countries in West Africa, much greater than the incidence of 38/100,000 reported here. From their model based on probabilities that a biting dog is rabid, that rabies develops in the absence of PEP, that victims receive PEP, the estimated bite incidence, and other factors, these authors estimate a per capita death rate of 0.98/100,000 for an annual total of ~200 annual rabies deaths for all of Cameroon [3]. The reporting of only four suspected cases during 2.5 years of surveillance in the West region, which encompasses approximately 10% of the population (20 million in 2010), suggests that many human rabies cases are still not being captured by current surveillance, despite the reinforcements put in place. We suspect that in Cameroon, as in other African countries, passive surveillance remains inefficient because it misses many bites and human rabies cases that occur in victims who never present to the healthcare system. Community ignorance may still be a factor, as well as the lack of effective treatments for symptomatic rabies disease, which can result in affected individuals choosing to die at home rather than in a clinic. Remaining needs are to increase PEP availability and to educate communities about the risks of rabies so that bite victims seek PEP immediately rather than waiting until symptoms appear.

Human rabies diagnosis in Cameroon and most rabies-endemic countries is based on clinical criteria rather than laboratory confirmation [25]. This can also contribute to inaccurate estimates of disease incidence and public health impact. The reinforced surveillance network enabled four clinically suspected cases of human rabies to be sampled and submitted for laboratory confirmation, only one of which was confirmed. These samples and the seven samples submitted from other regions are likely among the first human samples to be analyzed for rabies in Cameroon, and represent a step forward in human rabies surveillance. Of the three suspected cases in the study that were not confirmed, the two that died may have also been rabies cases because a negative result in this analysis, in which the sensitivity could be less than 100%, does not necessarily rule out disease [29]. Unfortunately, the symptoms of these cases were not available to support the clinical diagnoses because they were not recorded. It is unlikely that the third non-confirmed case was rabies because this patient survived. Although there were only four suspected cases, these results suggest that the specificity of the rabies diagnoses based on clinical criteria was, at best, 75%, and may need to be improved and should be accompanied by laboratory testing to confirm the diagnosis. Nevertheless, of the 11 suspected and confirmed human rabies cases reported nationwide, four (36%) were recorded from the included West region districts, despite the fact that these areas encompass only ~7% of the total population. This suggests that human rabies cases are also significantly underreported in the rest of the country.

As found in other countries with endemic canine rabies [2,5,30,31], exposures were frequent in children, with over 40% in persons ≤ 15 years of age, and mostly involved dogs. The delay between animal exposure and medical consultation was short, which suggests good public awareness of the risk of rabies in the West region. However, 703 exposures were considered to be at risk of rabies (or unknown) and only 428 (61%) of these cases received any PEP, leaving 275 (39%) at-risk exposure victims who received no PEP at all. Additionally, only one-half of those who started PEP were confirmed to have completed the vaccination series. Immediate administration of PEP is recommended by the WHO after animal bites or scratches, even in cases where the animal involved shows no sign of rabies, but can be interrupted if the animal is determined to be free of rabies [2]. The poor rates of PEP uptake and completion may be due to several reasons, including ignorance of the rabies risk, the relatively high cost of unsubsidized PEP in Cameroon, the costs and distances of the travel involved to return to the clinic for additional doses, and/or inability to leave work [30,32]. The cost of rabies vaccine in Cameroon is approximately 11.50€ per vial, thus total costs are 46€ for the Zagreb protocol and 57.50€ for the Essen protocol, either of which is a great financial burden in a country where the monthly minimum salary is around 55€. Although costs might be reduced with an intradermal administration protocol, this option is not currently being pursued in Cameroon due to the additional training of healthcare staff it requires. In the West region, victims might need to travel from 10 to 150 km from their residence to Bafoussam for PEP. However, this is an improvement because prior to the reinforcement project, the nearest ARC was in Yaoundé, which is 300 km from Bafoussam, the capital city of the West region. This highlights that further awareness campaigns and subsidization initiatives are needed to improve PEP accessibility in terms of cost and distribution.

Recent reports from the Philippines, Haiti, and Tanzania provide impressive examples of effective rabies surveillance and control programs in endemic countries [33–35]. In Haiti, active community bite investigations and comprehensive systematic passive animal rabies investigations, which were supported by the national ministries of agriculture and public health and the US Centers for Disease Control and Prevention, revealed a rabies burden that was much higher than previously reported [34]. In addition, many more at-risk exposures were discovered during bite investigations, suggesting that perhaps only 2/3 of bite victims seek healthcare treatment. Also in Haiti, an integrated bite case management program that counsels bite victims on the risk of rabies and appropriate PEP was found to increase healthcare-seeking behavior and PEP uptake [36]. In the Philippines, the Bohol Rabies Prevention and Elimination Project was developed that incorporated resources and funding from the national ministries of agriculture, public health and safety, education, environment, and legal affairs, as well as support from interior and local governments and international partners [33]. The project addressed local community involvement, dog population control, mass dog vaccinations, improved dog bite management, veterinary quarantines, and improved diagnostic capability, surveillance, and monitoring. As a result of the project, initiated in 2007, human rabies deaths in the region decreased from 10 cases per year in 2006 and 2007 to no cases in 2010. Laboratory-confirmed dog cases also dropped from 5 cases in 2007 to no cases in 2009. In Tanzania, rabies surveillance via a mobile phone network was found to be an effective means of providing accurate and timely animal exposure information to improve health care delivery and disease control activities and to better optimize distribution of the limited PEP supply [35].

However, these programs are much farther ahead than the enhanced surveillance program initiated in Cameroon, which is currently focused on instituting a system for collecting reliable data that can be used to establish the magnitude of the burden of disease and to improve PEP uptake. Nevertheless, these programs provide examples of how surveillance and control might be improved in Cameroon, once the burden of disease is established and the additional resources for control can be obtained.

Although animal rabies surveillance was not a major aspect of the reinforced surveillance and veterinary support was limited, seven animal samples were collected during veterinary follow-up investigations and submitted for rabies confirmation, of which four of the six dogs (67%) were confirmed positive. Despite the small number of samples, these results are comparable to dog rabies confirmation rates previously reported for Cameroon [8,9]. Of the 721 dogs that died during observational quarantine in the 1990−1999 period, 330 (46%) were confirmed positive [8], whereas a confirmation rate of 74% was obtained for 89 suspected rabid dogs sampled between 2010 and 2013 [9]. These results confirm that rabies is present in a substantial proportion of the dogs suspected of being rabid. Given that ~70% of the dogs investigated were confirmed rabid, it is likely that similar large proportion of the animals not investigated were also rabid.

One of the strengths of the program was that reinforcement was implemented at the level of the local health centers where it is expected that most exposure victims would seek initial treatment and PEP. In this way, the system was likely to capture a high proportion of the animal exposures that occurred in the catchment areas of these clinics. However, for approximately 30% of the exposures who did seek treatment or PEP, no CRFs were completed and the exposures were not actively reported to the District Health Authority but revealed only during weekly follow-ups performed by the CPC. Upon investigation, District Health Authority managers reported that CRFs were not completed for some exposures due to lack of sufficient personnel and high workloads. The failure to complete a CRF for an exposure was likely to be a random event so we do not expect that these exposures were any different from those documented in the CRFs. In addition, some sections of the CRFs were often incorrectly completed, notably the severity of exposure indicated by the WHO exposure classification [2]. For example, 7.2% of exposures involving bites and scratches were incorrectly recorded as WHO category I, which does not include these more severe types of injury (Table B in S1 Information). The signs and symptoms that could help determine rabies diagnostic accuracy were also not recorded in the CRF for several human rabies cases. In Cameroon, most people have a personal “hospital medical book” which they take to the hospital and is preferentially filled in by medical staff. When the patients leave the hospital, they also take their hospital medical book, which may have prevented the data from being entered in the CRF. To improve on these weaknesses in the surveillance network, strategies are needed for greater engagement of healthcare professionals at the local health centers and of veterinary services in the field. Ideally, additional healthcare and veterinary personnel are also needed, however, strategies that do not require additional resources, such as simplifying the reporting procedures or CRFs, could also be effective and stand a greater chance of being implemented.

The study also identified several weaknesses in animal rabies surveillance, as well as obstacles to its improvement. Suboptimal engagement from veterinary services was particularly apparent, as only 14% of the suspected rabid animals were investigated by a veterinarian subsequent to the potential rabies exposure. Although this was largely due to lack of sufficient human resources, as well as inadequate funding and facilities for housing and maintaining animals under observational quarantine, the surveillance system did not necessarily include direct contact between healthcare staff and veterinary services. In addition, animal owners are required to pay the costs of these services, which may include transportation costs for the veterinarian and/or the animal, and many refuse or are unable to do so. Such costs are a clear disincentive to veterinary follow-ups. Also, veterinarians in Cameroon often have to cover large populated districts alone and often spend much of their time on animal breeding activities as a source of income; they were not paid for collecting samples from animals suspected to be rabid. These factors, plus the strong market for dog meat (seen as a delicacy in much of Cameroon) [37], may also explain why very few animal samples were submitted for laboratory testing. Veterinary involvement in rabies surveillance might be improved by including direct communication between healthcare staff and veterinary services. However, without additional governmental support to increase veterinarian availability and services and to cover the costs of follow-up investigations and quarantines, it will be difficult to improve veterinarian engagement in animal rabies surveillance.

A limitation to this study was that few performance measures could be calculated to assess how well the reinforcements improved existing surveillance of human and animal cases. Reasons for this included the small number of samples submitted for rabies confirmation and the limited available data from previous surveillance activities in the West region. Nevertheless, the reinforcements greatly improved animal exposure reporting and provided improved training, coordination, sampling, and laboratory confirmation of rabies. This is the first report of active surveillance in Cameroon.

Although the reinforced surveillance network may have reduced underreporting of animal exposures in the West region of Cameroon, its findings may still underestimate the burdens of animal exposures and rabies cases in the region, compared to current modeling estimates. To help address this, integration of the nine remaining West region districts into the surveillance network is planned and efforts to improve collaboration between healthcare and veterinary services and completion of notification forms are underway. The successes of the West region’s improved surveillance network have contributed to a new government strategy to build an improved nationwide surveillance system, in which rabies surveillance will be prioritized, as part of a national zoonosis program [38].

## Supporting information

S1 Information Supporting tables and figures. (PDF)

S1 Data Raw data for 2014−2015. (PDF)

S2 Data Raw data for 2014−2016. (PDF)

S3 Data Population data for the West region of Cameroon. (PDF)
