# Supplementary material for: Improving systematic rabies surveillance in Cameroon: A pilot initiative and results for 2014-2016
Source: PLoS Negl Trop Dis. 2018 Sep 6;12(9):e0006597. doi: 10.1371/journal.pntd.0006597 (PMC6126802; doi:10.1371/journal.pntd.0006597)
Supplement: S1 Information — (PDF) [file pntd.0006597.s001.pdf]

S1 Supporting Information

Sofeu et al. Improving systematic rabies surveillance in Cameroon: a pilot initiative and results for 2014−2016

Figure A: Animal bites notifications per epidemiological week from 2014 to June 2016 in the West region, Cameroon.

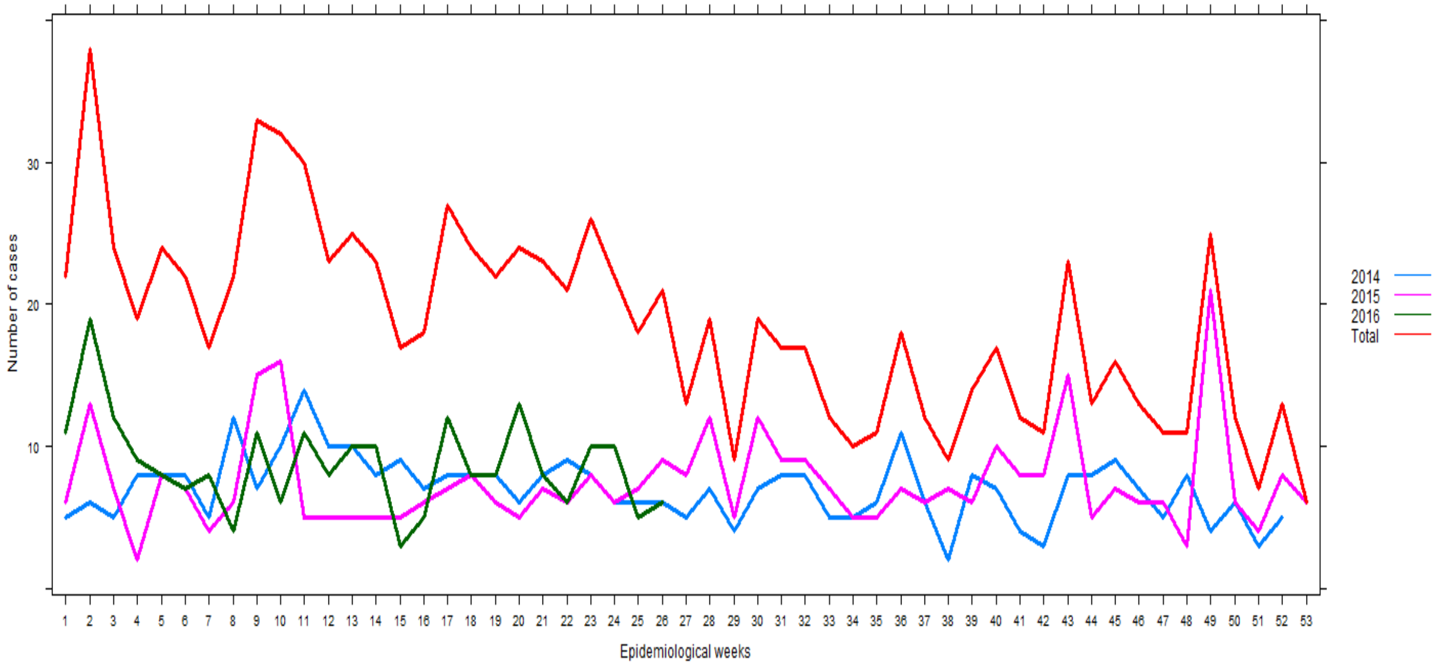

Table A: Lesion or wound description for animal exposures from 2014 to June 2016 week 26, West region, Cameroon.

	Total	
**Characteristics**	**N=925**	**%**
Contact type		
Bite	845	91.4
Scratches	68	7.4
Licking	2	0.2
Missing	10	1.1
Contact site		
Cutaneous	822	88.9
mucous	6	0.6
Missing	97	10.5
WHO category		
I	68	7.4
II	579	62.6
III	139	15.0
Missing	139	15.0
Interposition of clothes		
No	490	53.0
Yes	339	36.6
Missing	96	10.4

Table B: Non-specific post-exposure treatments from 2014 to June 2016 week 26, West region, Cameroon.

	Total	
**Characteristics**	**N=925**	**%**
Anti-tetanus vaccine		
No	536	57.9
Yes	198	21.4
Missing	191	20.6
Tetanus prevention status		
Up to date	122	13.2
Primary vaccination	78	8.4
Booster	4	0.4
Missing	721	77.9
Administration of anti-tetanus serum		
No	161	17.4
Yes	669	72.3
Missing	95	10.3
Use of antibiotics treatment		
No	204	22.1
Yes	624	67.5
Missing	97	10.5
