# Supplementary material for: Improving systematic rabies surveillance in Cameroon: A pilot initiative and results for 2014-2016
Source: PLoS Negl Trop Dis. 2018 Sep 6;12(9):e0006597. doi: 10.1371/journal.pntd.0006597 (PMC6126802; doi:10.1371/journal.pntd.0006597)
Supplement: S1 Data — (PDF) [file pntd.0006597.s002.pdf]

Table 1

	Animal rabies suspicion nationwide			
Samples received (heads)	**2014**		**2015**	
	n=31	Positive (%)	n=21	Positive (%)
**Dog**	30	20 (66.7)	20	9 (45.0)
**Cat**			1	0
**Pig**	1	0		

Table 2

	Animal rabies suspicion in the West region			
Samples received (heads)	**2014**		**2015**	
	n=6	Positive (%)	n=1	Positive (%)
**Dog**	5	4 (80.0)	1	0
**Pig**	1	0		

Table 3

Clinical suspicion of rabies nationwide			
2014		**2015**	
**n**	Died	n	Died
**15**	14	15	14

Table 4

	Human rabies suspicion Nationwide			
Samples received	**2014**		**2015**	
	n	Positive (%)	n	Positive (%)
**Saliva/skin biopsy**	7	2(28.6)	**4**	**2 (50.0)**

Table 5

	Human rabies suspicion West region			
Samples received	**2014**		**2015**	
	n	Positive (%)	n	Positive (%)
**Saliva/skin biopsy**	1	0	3	1 (33.3)

Table 6

Human rabies in West region					
Type of sample	**Age**	**Gender**	**Symptoms/signs**	**Result**	**Outcome**
**Saliva/skin biopsy**	8	M	fièvre, agitation, hydrophobie, exposition	Positive	Died
**skin biopsy**	8	M		Negative	Died
**skin biopsy**	5	M		Negative	Died
**Saliva/skin biopsy**	15	M		Negative	Alived
