# Supplementary material for: Improving systematic rabies surveillance in Cameroon: A pilot initiative and results for 2014-2016
Source: PLoS Negl Trop Dis. 2018 Sep 6;12(9):e0006597. doi: 10.1371/journal.pntd.0006597 (PMC6126802; doi:10.1371/journal.pntd.0006597)
Supplement: S2 Data — (PDF) [file pntd.0006597.s003.pdf]

Mise en place d'un système de recueil d'informations épidémiologiques de base sur la rage dans la région de l'Ouest au Cameroun

Données considérées : Semaine épidémiologique N°1 année 2014 à la semaine N°26 de l'année 2016

1. Brief synthesis of the epidemiological situation

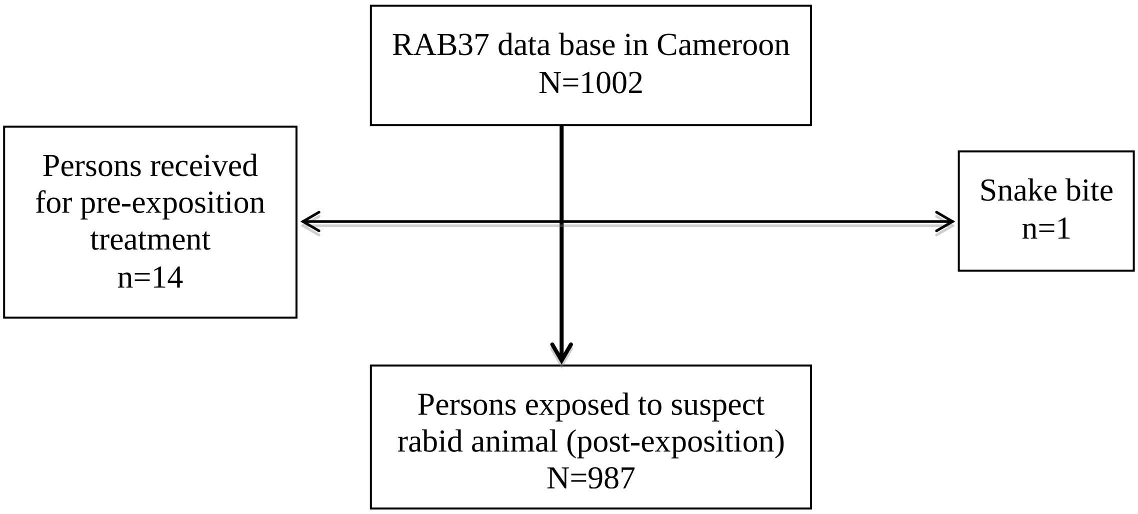

Table1: Distribution of suspected rabid animal bites cases reported between the first epidemiological week of 2014 and week 26 of 2016 per health district in the Western region of Cameroon, RAB37 CPC-SANOFI PASTEUR

	Years			Total	
**District**	**2014**	**2015**	**2016**	**N**	**%**
Bafang	8	5	24	37	3.7
Baham	11	4	7	22	2.2
Bamendjou	13	5	10	28	2.8
Bandja	3	3	0	6	0.6
Bandjoun	20	11	2	33	3.3
Bangangté	44	30	12	86	8.7
Bangourain	3	3	0	6	0.6
Batcham	5	2	2	9	0.9
Dschang	19	13	23	55	5.6
Foumban	10	10	16	36	3.6
Foumbot	6	5	4	15	1.5
Galim	3	2	0	5	0.5
Kekem	13	11	0	24	2.4
Kouoptamo	0	2	1	3	0.3
Malentouen	5	3	0	8	0.8
Mbouda	30	31	19	80	8.1
Mifi	166	217	100	483	48.9
Penka michel	3	30	8	41	4.2
Out of the west region	0	1	0	1	0.1
Not indicated	2	7	0	9	0.9
Total (all districts)	364	395	228	987	100
Total (RAB37 districts)	337	367	221	925	93.8

Districts involved in the RAB37 pilot study

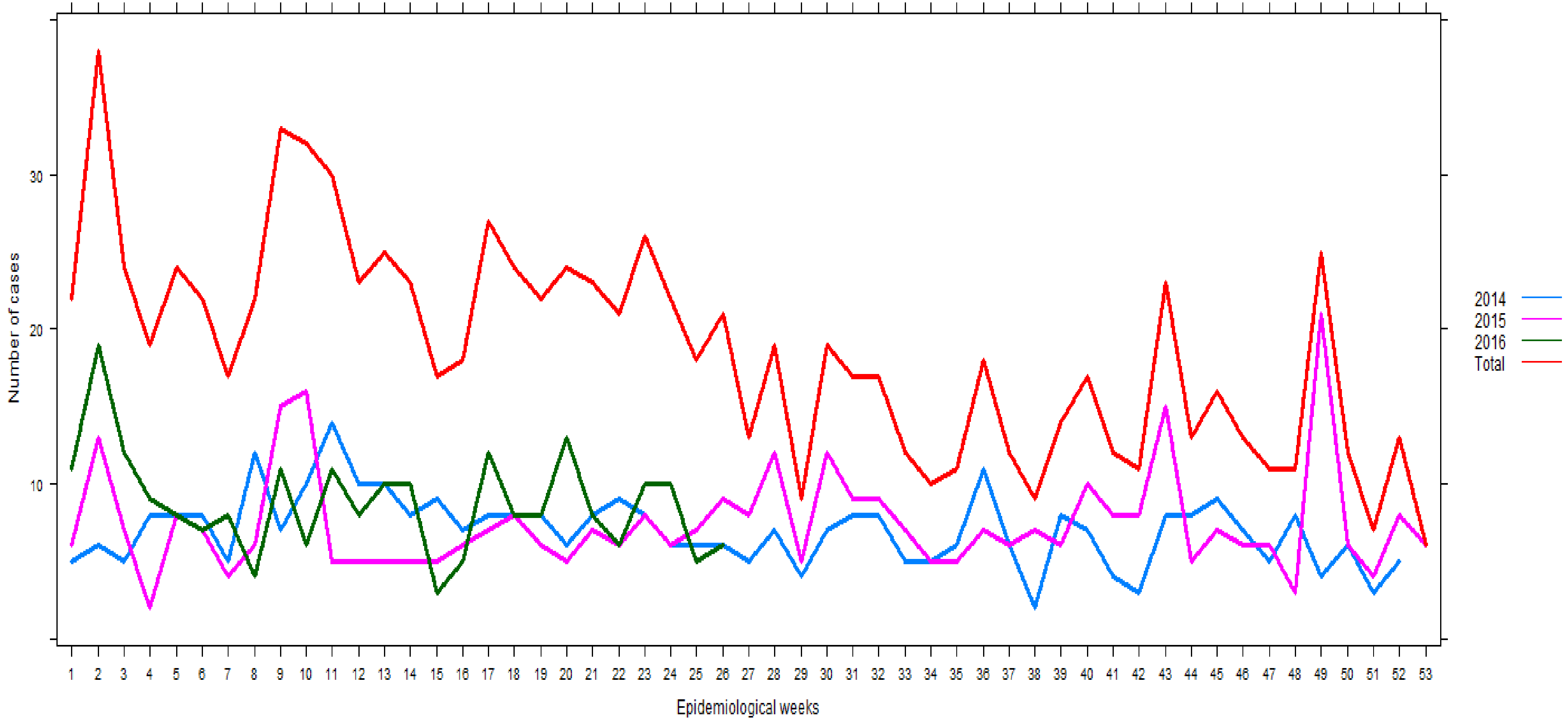

Figure 1: Suspected Rabid animal bites notification per epidemiological week from 2014 to June 2016 in the West region of Cameroon, RAB37 CPC-SANOFI PASTEUR

2. Animal involved in the bite of human

Table 2: Distribution of animals involved in the bite of human from 2014 to week 26 June 2016 in the West region of Cameroon, RAB37 CPC-SANOFI

	2014		2015		2016		Total	
**Animals**	**n**	**%**	**n**	**%**	**n**	**%**	**N**	**%**
Dog	239	93.4	305	93.3	174	93.5	**718**	**93.4**
Cat	5	2.0	11	3.4	6	3.2	**22**	**2.9**
Mouse	1	0.4	4	1.2	1	0.5	**6**	**0.8**
Pork	2	0.8	7	2.1	3	1.6	**12**	**1.6**
Domestic monkey	3	1.2	0	0.0	0	0.0	**3**	**0.4**
Domestic animal	2	0.8	0	0.0	0	0.0	**2**	**0.3**
Donkey	1	0.4	0	0.0	0	0.0	**1**	**0.1**
unknown	3	1.2	0	0.0	2	1.1	**5**	**0.7**
**Total**	**256**	**33.3**	**327**	**42.5**	**186**	**24.2**	**769**	**100.0**

Table 2 taking into consideration missing data

	2014		2015		2016		Total	
**Animals**	**n**	**%**	**n**	**%**	**n**	**%**	**N**	**%**
Dog	239	70.9	305	83.1	174	78.7	**718**	**77.6**
Cat	5	1.5	11	3.0	6	2.7	**22**	**2.4**
Mouse	1	0.3	4	1.1	1	0.5	**6**	**0.6**
Pork	2	0.6	7	1.9	3	1.4	**12**	**1.3**
Domestic monkey	3	0.9	0	0.0	0	0.0	**3**	**0.3**
Domestic animal	2	0.6	0	0.0	0	0.0	**2**	**0.2**
Donkey	1	0.3	0	0.0	0	0.0	**1**	**0.1**
Unknown	3	0.9	0	0.0	2	0.9	**5**	**0.5**
Missing	81	24.0	40	10.9	35	15.8	**156**	**16.9**
**Total**	337	36.4	367	39.7	221	23.9	**925**	**100**

3. Characteristics of people bitten by suspect rabid animals.

a) Patient identification, reason and consultation history

Table 3: Characteristics of people people bitten by suspect rabid animals from 2014 week 26to June 2016, West region, RAB37 CPC-SANOFI PASTEUR

	Total	
Characteristics	**N=925**	**%**
Sex		
Female	498	53.8
Male	416	45.0
Missing	11	1.2
Age: Median [IQR], in years	20[9-42]	
Missing	6	0.6
Delay from animal bite to medical consultation: Median [EIQ], in days	1[0-3]	
Missing	102	11.0
Previous history of suspect rabid animals		
No	822	88.9
Yes	73	7.9
Missing	30	3.2
Reason of consultation		
Contact with suspect rabid animal	924	99.9
Missing	1	0.1

b) Lesion or wound in human after animal contact

Table 4: Lesion or wound description in people bitten by suspect rabid animal from 2014 to June 2016, West region, RAB37 CPC-SANOFI PASTEUR

	**Total**	
**Characteristics**	**N=925**	**%**
Contact type		
Bite	845	91.4
Scratches	68	7.4
Licking	2	0.2
Missing	10	1.1
Contact site		
Cutaneous	822	88.9
mucous	6	0.6
Missing	97	10.5
WHO category		
I	68	7.4
II	579	62.6
III	139	15.0
Missing	139	15.0
Interposition of clothes		
No	490	53.0
Yes	339	36.6
Missing	96	10.4

Description of "lesion or wound" with Who category = I or missing (with the following description, we can review the WHO category in the above Table 4)

	WHO category			
	I		Missing data	
Type contact	N=68	%	N=139	%
Scratches	13	19.1	12	9.3
Licking	1	1.5	1	0.8
Bite	54	79.4	116	89.9
Total	68	100	129	92.8
Contact site				
Cutaneous	63	100	107	97.3
Mucous	0	0	3	2.7
Total	63	92.6	110	79.1

c) Description of suspect rabid animals by nurses and physicians

Table 5: Characteristics of suspect rabid animals from 2014 to June 2016, West region, RAB37 CPC-SANOFI PASTEUR

	Total	
**Characteristics**	**N=925**	**%**
Suspect rabid animal		
No	222	24.0
Yes	637	68.9
Missing	66	7.1
Known animal/ known animal owner		
No	260	28.1
Yes	592	64.0
Missing	73	7.9
Vital status of the animal at the time of consultation		
alive	609	65.8
disappeared	207	22.4
killed	29	3.1
deceased	11	1.2
Missing	69	7.5
animal vaccinated		
No	458	49.5
Yes	117	12.6
Missing	350	37.8
animal surveillance by veterinarian		
No	605	65.4
Yes	133	14.4
Missing	187	20.2
Laboratory diagnosis of rabies		
Negative	2	0.2
Positive	2	0.2
Not practised	650	70.3
Missing	271	29.3

	Suspect rabid animal			
Treatment Received	No	%	Yes	%
No	132	59.5	230	36.1
Yes	90	40.5	407	63.9
Total	222	25.8	637	74.2

d) Non specific treatments administered to people bitten by suspect rabid animals

Table 6 : Description of Non specific post exposure treatments from 2014 to June 2016, West region, RAB37 CPC-SANOFI PASTEUR week 26

	Total	
**Characteristics**	**N=925**	**%**
Anti-tetanus vaccine		
No	536	57.9
Yes	198	21.4
Missing	191	20.6
Tetanus prevention status		
Up to date	122	13.2
Primary vaccination	78	8.4
Booster	4	0.4
Missing	721	77.9
Administration of anti-tetanus serum		
No	161	17.4
Yes	669	72.3
Missing	95	10.3
Use of antibiotics treatment		
No	204	22.1
Yes	624	67.5
Missing	97	10.5

e) Specific rabies treatments

Table 7: Post exposure treatment from first epidemiological week 2014 to week 26 of 2016, Western region, RAB37 CPC-SANOFI

	Total	
**Characteristics**	**N=925**	**%**
Post exposition treatment (PET) administered		
Yes	518	56
No	407	44
PET completed[$,*]		
Yes	282	54.4
Missing	236	45.6
PET Protocol [$]		
ZAGREB	496	95.8
ESSEN	15	2.9
Missing	7	1.3
Vaccine used[$]		
Verorab®	496	95.7
Antirabique (without precision)	5	1.0
Missing	17	3.3
Side effect[$]		
No	342	66.0
Missing	176	34.0

[$]Calculated percentage in relation to people treated
*compilation of data from different pilot sites registers is ongoing. The result would certainly change what we have in the database to date

Table 8: Post exposition treatment and WHO category/clinical suspicion of rabies, RAB37 CPC-SANOFI

	WHO category							
	1		2		3		Missing data	
	n=68	%	n=579	%	n=139	%	n=139	%
Post Exposure Treatment								
No	41	60.3	170	29.4	87	62.6	109	78.4
Yes	27	39.7	409	70.6	52	37.4	30	21.6

	Suspect rabid animal					
	No		Yes		Missing data	
Post exposure treatment	n=222	%	n=637	%	n=66	%
No	132	59.5	230	36.1	45	68.2
Yes	90	40.5	407	63.9	21	31.8
