# Supplementary material for: Improving systematic rabies surveillance in Cameroon: A pilot initiative and results for 2014-2016
Source: PLoS Negl Trop Dis. 2018 Sep 6;12(9):e0006597. doi: 10.1371/journal.pntd.0006597 (PMC6126802; doi:10.1371/journal.pntd.0006597)
Supplement: S3 Data — (PDF) [file pntd.0006597.s004.pdf]

RAB37 results 3

Tableau 1 : Population de la région de l'Ouest selon les districts de santé - 2011

	District de santé	Population
1	BAFANG	92 651
2	BAHAM	49 823
3	BAMENDJOU	48 437
4	BANDJA	37 022
5	BANDJOUN	111 948
6	BANGANGTE	110 979
7	BANGOURAIN	28 970
8	BATCHAM	88 069
9	DSCHANG	206 652
10	FOUMBAN	166 411
11	FOUMBOT	57 779
12	GALIM	40 647
13	KEKEM	38 105
14	KOUOPTAMO	41 830
15	MALENTOUEN	69 150
16	MASSANGAM	21 783
17	MBOUDA	244 846
18	MIFI	247 925
19	PENKA MICHEL	86 183
20	SANTCHOU	21 438
	**TOTAL**	**1 810 648**
